# Wear Degree Quantification of Pin Connections Using Parameter-Based Analyses of Acoustic Emissions

**DOI:** 10.3390/s18103503

**Published:** 2018-10-17

**Authors:** Jingkai Wang, Linsheng Huo, Chunguang Liu, Gangbing Song

**Affiliations:** 1 State Key Laboratory of Coastal and Offshore Engineering, Dalian University of Technology, Dalian 116024, China; wjk@mail.dlut.edu.cn (J.W.); liucg@dlut.edu.cn (C.L.); 2 Smart Materials and Structures Laboratory, Department of Mechanical Engineering, University of Houston, Houston, TX 77204, USA

**Keywords:** acoustic emission (AE), *b*-value method, quantification of wear degree, wear frequency spectrum, wear of pin connections

## Abstract

Pin connections are commonly used in many engineering fields, and continuous operation may cause severe wear on the pins and may lead to their eventual fracture, if undetected. However, a reliable nonintrusive real-time method to monitor the wear of pin connections is yet to be developed. In this paper, acoustic emission (AE)-based parametric analysis methods, including the logarithm of the cumulative energy (LAE), the logarithm of the slope of cumulative energy (LSCE), the *b*-value method, the *Ib*-value method, and the fast Fourier transformation (FFT), were developed to quantify the wear degree of pin connections. The *b*-value method offers a criterion to quickly judge whether severe wear occurs on a pin connection. To assist the research, an experimental apparatus to accelerate wear test of pin connections was designed and fabricated. The AE sensor, mounted on the test apparatus in a nondestructive manner, is capable of real-time monitoring. The micrographs of the wear of pins, and the surface roughness of pins, verified that the values of the max LAE and the max LSCE became larger as the wear degree of pin connections increased, which means different values of the max LAE and the max LSCE can reflect different wear degree of pin connections. Meanwhile, the results of the micrographs and surface roughness confirmed that the *b*-value is an effective method to identify severe wear, and the value “1” can be used as a criterion to detect severe damage in different structures. Furthermore, the results of spectrum analysis in the low frequency range showed that the wear frequency was concentrated in the range of 0.01 to 0.02 MHz for the pin connection. This study demonstrated that these methods, developed based on acoustic emission technique, can be utilized in quantifying the wear degree of pin connections in a nondestructive way.

## 1. Introduction

Pin connections have been widely applied in many engineering fields [1,2]. Compared with other types of connection forms, pin connections have the advantages of low cost, simplicity, and facilitation of disassembly for repair [3]. However, continuous operation may cause damage (such as severe wear, shear fracture, and others) on the pins, which may result in their eventual fracture if undetected [4,5]. Traditional inspection methods depend on human experience, and cannot quantify the damage of pin connections [6,7]. Many researchers have studied the mechanical properties of pin connections. Bridge et al. [8] studied the mechanical properties of pins by examining the influence of different parameters of pins, and proposed modifications to previous design procedures. Aktas et al. [9] researched the effect of sea water on pinned joints of glass fiber composite materials, and found the failure distance of pin displacement increased with the increase of immersing period. Liang et al. [4,10] used Piezoelectric patch to monitor the load conditions of pin connections. Li et al. [5] applied wavelet transform to monitor the health conditions under different load levels, and found that the energy of the transmitted signal increased with the load on the pin connections. Obviously, these studies focused on monitoring the load condition of pin connections. However, an effective real-time monitoring method, to quantify the wear degree of pin connections and to set a criterion to judge whether severe wear occurs, is yet to be developed.

In the past decades, real-time monitoring systems [11,12,13,14,15,16,17], especially in the area of structural health monitoring (SHM) [18,19,20,21], have been developed. The commonly used methods in these real-time monitoring systems include ultrasonic guided wave method [22,23], active thermography [23,24,25], eddy current method [26,27], electromechanical impedance [28,29,30], and others [31,32]. Among these methods, acoustic emission (AE) technique [33,34,35,36], a passive monitoring technique that detects the damage by analyzing the elastic stress wave generated as a result of deformation and fracture in materials, is appropriate to monitor the wear process of pin connections. Compared with other nondestructive evaluation methods, it is more sensitive and less susceptible to complex structural geometry [37], therefore, the AE technique has been widely used in different fields, including civil engineering [38,39], especially bridge engineering [40,41], mechanical engineering [42,43,44], and rock mechanics [45,46,47]. In civil engineering, Abouhussien et al. [48] exploited acoustic emission (AE) monitoring to classify the stages of bond deterioration under pull-out tests, and proposed damage classification charts based on the intensity analysis. Aldahdooh et al. [49] classified the types of cracks (flexural or shear cracks) of several types of reinforced concrete (RC) beams subjected to four-point bending by acoustic emission technique. In mechanical engineering, Kulandaivelu et al. [50] found the AE technique was sensitive to wear signals above 200 KHz of a single point cutting tool in turning. Benabdallah et al. [51] found the good relationship between RMS (Root Mean Square) and the friction coefficient in the sliding contact. Hase et al. [52] confirmed that the frequency of adhesive wear is larger than abrasive wear. Furthermore, taking its advantage of high sensitivity, researchers applied the AE technique to investigate bubble formation during the boiling process [33,53].

To improve sensibility of the AE technique and extend its fields of application, various data processing methods, such as wavelet packet transform [54,55], neural networks [56,57], genetic algorithms [58], deep learning [59,60,61], and clustering methods [62,63], were proposed. In addition to these methods, Kurz et al. [64] introduced Akaike information criterion (AIC) to the analysis of AE signals for picking signals accurately, and Schechinger et al. [65] applied this method to monitoring the deterioration progress of a reinforced concrete beam, under loading, with satisfactory results.

Compared with the above methods, the *b*-value method, which was developed by Gutenberg and Richter [66] in 1944, is a simple [40,67] and an effective method to deal with AE data. Many researchers applied this method to the study of the fracture mechanics of materials [68,69]. For example, Colombo et al. [67] applied *b*-value to analyze AE signals from reinforced concrete failure in experiments. Schumacher et al. [40] found that the minimum *b*-value analysis had the potential to estimate the load levels on operating RC bridge girders.

This paper explores the feasibility of quantifying the wear degree of pin connections and setting a criterion of severe wear. To facilitate the research, an experimental apparatus to accelerate wear test of a pin connection was designed and fabricated, and accelerated wear tests of pin connections were conducted. Signals were collected by AE sensors, which were mounted on the test apparatus in a nondestructive way. Three parameter-based methods were used to analyze these AE signals: the max logarithm of the cumulative energy (LAE) and the logarithm of the slope of cumulative energy (LSCE) were used to quantify the wear degree of pin connections; the *b*-value method and the *Ib*-value method were applied to distinguish the wear degree of pin connections; and fast Fourier transformation (FFT) was used to analyze the wear frequency spectrum in the low frequency range of pin connections. The experimental results showed that different values of the maximum of LAE and the maximum of LSCE reflected different wear degree. Based on which, experimental data were analyzed, and the four specimens can be divided into three groups: the severe wear group, the moderate wear group, and the slight wear group. Furthermore, the *b-*value method is an effective method to offer a criterion to quickly judge if the wear status of a pin connection is severe or not. In addition, spectrum analysis of AE signals demonstrated that the main wear frequency in the low frequency range decreases with the increase wear degree.

## 2. Materials and Methods

### 2.1. b-Value Method

In earthquake seismology, the numbers of events of larger magnitude is less than the events of smaller magnitude; this phenomenon can be quantified by the Gutenberg–Richter relationship [66], and the equation is as follows [67]:

(1) $$\log_{10}N=a-bM,$$

where *M* is the Richter magnitude of earthquakes; *N* is incremental frequency (i.e., the number of earthquake with magnitude greater than *M*); and *a* and *b* are coefficients [67]. There is a linear relationship between the logarithm value of incremental frequency and the Richter magnitude of earthquakes in the formula. Obviously, *b* is the slope, and reflects the proportion of low magnitude earthquakes in all earthquakes. Therefore, *b* will decrease with the increase of the number of larger magnitude earthquakes. The coefficient *b* is the *b*-value, whose intermediate increases firstly, and is then followed by a decrease in the months to weeks before an earthquake [70,71].

Shiotani [68,69] and Rao [72] assumed the same principle could be applied to the AE to study the cumulative frequency-magnitude relationship, and to reflect the damage characteristics during the rock fracture process. They showed the *b*-value equation adapts to AE method as

(2) log10N=a−b(AdB20),

where *N* is the incremental frequency (i.e., the number of AE signal with amplitude greater than the threshold); *a* and *b* are coefficients; and *A_dB_* is the amplitude of AE signals, which is the maximum AE signal excursion during an AE hit, and is defined as

(3) AdB=20log(Vmax1μv)−P,

where *V_max_* is the maximum voltage of an AE hit; and *P* is the preamplifier gain in dB. *N* and *A_dB_* can be directly obtained from the AE device. Therefore, *b*-value can be calculated from AE parameters by Equation (2). Furthermore, Shiotani proposed the improved *b*-value (*Ib*-value), which is defined as

(4) $$\;Ib=\;\frac{\log_{10}N\left(\mu -\alpha_{1}\sigma \right)-\log_{10}N\left(\mu -\alpha_{2}\sigma \right)}{\left(\alpha_{1}+\alpha_{2}\right)\sigma }\;,\;$$

where μ is the mean amplitude; σ is the standard deviation; *N* is the incremental frequency (i.e., the number of AE signal with amplitude greater than the threshold); $\alpha_{1}$ is the coefficient related to the smaller amplitude; and $\alpha_{2}$ is related to the fracture level. The feasibility of applying the *Ib*-value method to monitor wear degree of pin connections will be studied in the future. Following this, the physical acoustics corporation (PAC) proposed Equation (5) to calculate the *Ib*-value:

(5) $$\;Ib=\;\frac{\log_{10}N_{1}-\log_{10}N_{2}}{a_{2}-a_{1}},\;$$

(6) $$a_{1}=\mu -\alpha_{1}\sigma ,$$

(7) $$a_{2}=\mu +\alpha_{2}\sigma ,$$

where *N*_1_ and *N*_2_ represent the lower amplitude limit and upper amplitude limit of each amplitude distribution graph, respectively; μ is the mean amplitude; and σ is the standard deviation. Rao [72] compared the results of the *Ib*-value (Shiotani) and the *Ib*-value (PAC) in rock fractures, and found they have same tendency, however, the tendency of *Ib*-value (PAC) is closer to that of *b*-value when $\alpha_{1}$ = $\alpha_{2}$ = 1. Therefore, this paper calculates *Ib*-value by using Equations (5)–(7), and $\alpha_{1}$ = $\alpha_{2}$ = 1.

### 2.2. Test Equipment and Procedures

**Figure 1:**
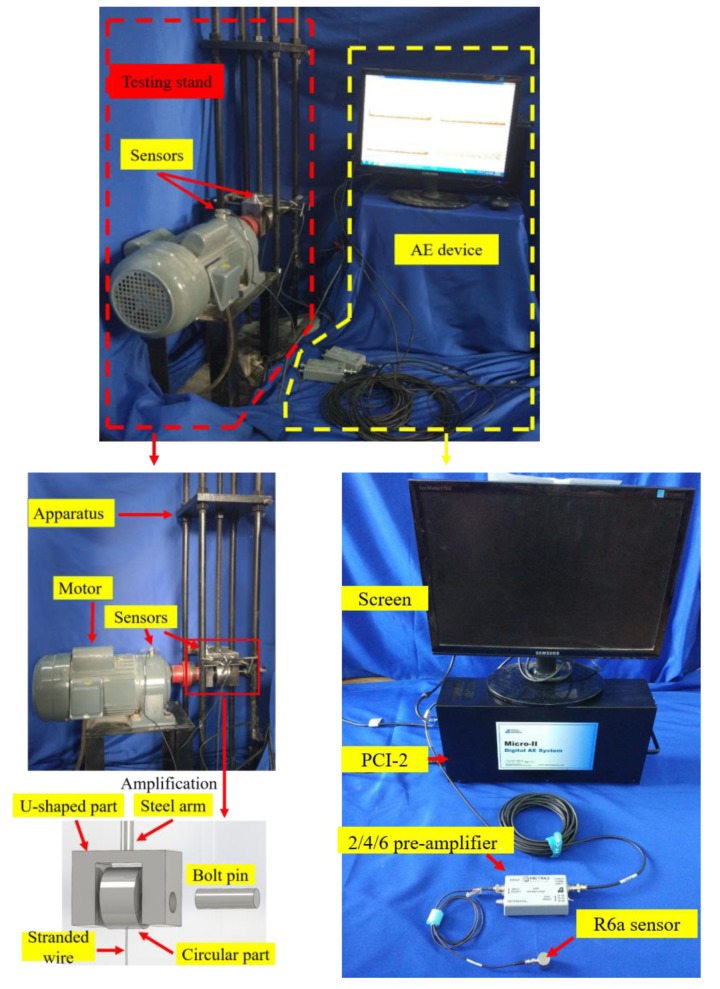
Photos of experimental apparatus.

In this paper, research was performed by using the experimental setup as shown in Figure 1. The experiment setup consists of a testing stand and an AE system. The core parts of the testing stand include the U-shaped part, the circular part, and the pin. The circular part was connected to bottom steel plate of the testing stand by stranded steel cable, and the U-shaped part was fixed by a steel arm which was connected to top steel plate by a bolt. The U-shaped part and the circular part were connected by a pin, and the connection between them has a tight fit. The lubricant used in the experiment was No. 3 lithium base grease, which is made by lithium hydroxy fatty acid, medium viscosity mineral lubricating oil, antioxidants, and others. It has been widely used as the lubricant in many rotating machines, such as water pump, blowing machine, motor, and others. The material of the core parts is high speed steel, and the chemical compositions of specimens are summarized in Table 1. Four pins, as specimens, were used in this experiment, and the same tested condition was used. A motor was used to rotate the pin to accelerate the wear of the pin connection in experiments. To balance the authenticity of simulation and the speed of experiment, the rotational speed of the motor was set to 30 rpm. The testing stand and the motor were fixed on a steel plate. The test duration was controlled to around 10 h. However, when severe wear occurred, the friction between the pin and the U-shaped part will dramatically increase and generate high-level noises. In addition, the increased friction force will cause the U-shaped part to rotate with the pin, which may result in breaking the stranded steel cable and damaging the experimental setup. To avoid such a situation, the test will be stopped before the stranded cable breaks. Basically, by listening to the noise, which is closely related to the AE, and observing the motion of the U-shaped part, we can judge the severity of the wear.

### 2.3. AE Test Equipment and Measurement Equipment

A PCI-2 8-channel AE system (from Physical Acoustic Corporation, Princeton, NJ, USA) was used in this experiment. The AE sensor applied in the experiment was R6a, whose frequency is from 35 to 100 KHz, and whose resonance frequency is 55 KHz. The results of this experiment confirmed that AE signals collected by R6a sensor can reflect the change of wear degree. Furthermore, the R6a sensor has high sensibility in the low frequency range, and can be used to study the wear frequency in low frequency ranges. Two AE sensors were used in this experiment, in a nondestructive way, to real-time monitor the wear degree of pin connections. One was mounted on the upper surface of the U-shaped part, and the other was mounted on the upper surface of the motor to collect the AE signals of motor, as shown in Figure 1. Meanwhile, the pre-amplifier was type 2/4/6, and its gain was set to 40 dB. In the wear process of pin connections, interference signal is mainly from background noise, motor noise, and the noise caused by reflection of AE signals. For background noise, since its amplitude is low, setting a threshold can filter it out. Usually, the threshold is 45 dB, which means that only these AE signals whose amplitude is over 45 dB can be collected by the PCI-2 system. For motor noise, a sensor was mounted on the upper surface of motor for the measurement. Motor noise can be filtered out by comparing the AE signals from the wear process of pins and that from motor. For the noise caused by reflection of AE signals, front-end filters were used to reduce reflections by setting values of PDT (peak definition time: ensures correct identification of the signal peak for risetime and peak amplitude measurements), HDT (hit definition time: ensures that each AE signal from the structure is reported as one and only one hit), and HLT (hit lockout time: inhibits the measurement of signals after the hit stored to avoid measuring reflection). In this experiment, PDT, HDT, and HLT were set to 300, 600, and 1000 µs. The connecting of the system is shown in Figure 1.

**Figure 2:**
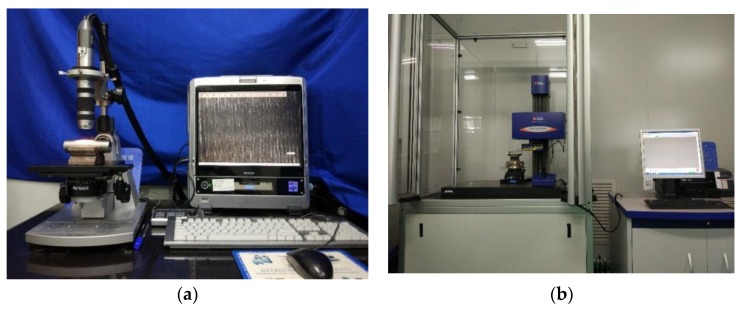
Measurement equipment: (**a**) VHX-600E digital microscope; (**b**) PGI 840 roughmeter.

To verify the results of AE technique, the VHX-600E digital (from Keyence, Osaka, Japan) microscope was applied to observe the worn surface of pins (as shown in Figure 2a), and the PGI 840 roughmeter (from Taylor Hobson, Leicester, UK) was applied to measure the surface roughness of pins (as shown in Figure 2b). The detailed parameters of the microscope and the roughmeter are summarized in Table 2 and Table 3, respectively. Furthermore, in the process of measuring roughness, a control specimen, in which no wear occurred, and four specimens, were measured. Since the measurement range of roughmeter is a line, this experiment will measure 8 lines in wear areas of four specimens, and the length of each line is 8 mm.

## 3. Experimental Results and AE Parameter-Based Analyses

### 3.1. Features of AE Parameters

**Figure 3:**
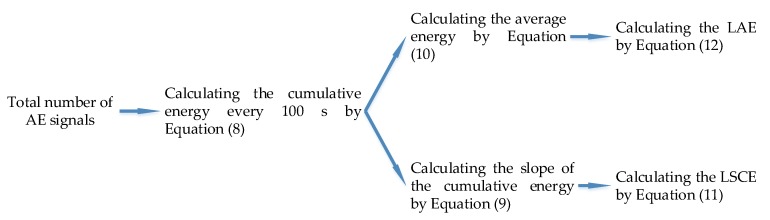
The flow chart to calculate the average energy and the slope of cumulative energy.

Energy is one of the most important AE parameters, and it is the integral of the rectified voltage signal over the duration of the AE hit (or waveform). Compared with amplitude (the maximum AE signal excursion during an AE hit), energy has better sensitivity and a larger range. However, it is difficult to judge whether the maximum energy value has been influenced by noise. To reduce these interference factors, the average energy and the slope of cumulative energy will be used in this paper. The average energy can also reduce the negative impact of different duration of four specimens by dividing the duration. The related equations are as follows:

(8) $$\;Q\left(T_{i}\right)=\;\overset{n}{\underset{j=1}{\sum }}e_{j},\;$$

(9) $$\;S\left(T_{i}\right)=\;\frac{Q\left(T_{i+1}\right)-Q\left(T_{i}\right)}{T_{i+1}-\;T_{i}},\;$$

(10) $$\;M\left(T_{i}\right)=\;\frac{Q\left(T_{i}\right)}{T_{i}},\;$$

(11) $$LSCE\left(T_{i}\right)=\log_{10}S(T_{i}),$$

(12) $$LAE\left(T_{i}\right)=\log_{10}M(T_{i}),$$

where *Q*(*T_i_*) is the cumulative energy in *T_i_* s; *e_j_* is the *j*th hit; *n* is the number of hits in *T_i_* s; *S*(*T_i_*) is the slope of cumulative energy at the *T_i_* s; *M*(*T_i_*) is the average energy in *T_i_* s; *LSCE*(*T_i_*) is the logarithm of the slope of cumulative energy (LSCE) at the *T_i_* s; and *LAE*(*T_i_*) is the logarithm of the average energy (LAE) at the *T_i_* s. Furthermore, in this paper, since the number of data units from one experiment is over 1000,000, to balance the computational time and the accuracy, the cumulative energy and the slope of cumulative energy will be counted every 100 s, which means that, in Equation (11), the value of denominator is 100. The detailed process is illustrated in Figure 3.

Furthermore, the range of energy collected by PCI-2 equipment is from 0 to 65,535 aJ. Results show that the energy values reach the maximum energy of system in this experiment, which means that there may exist some AE signals whose energy value is larger than the max energy of system in this experiment. However, the results of this paper confirm that the wear degree can be quantified in the range of 0 to 65,535 aJ.

**Figure 4:**
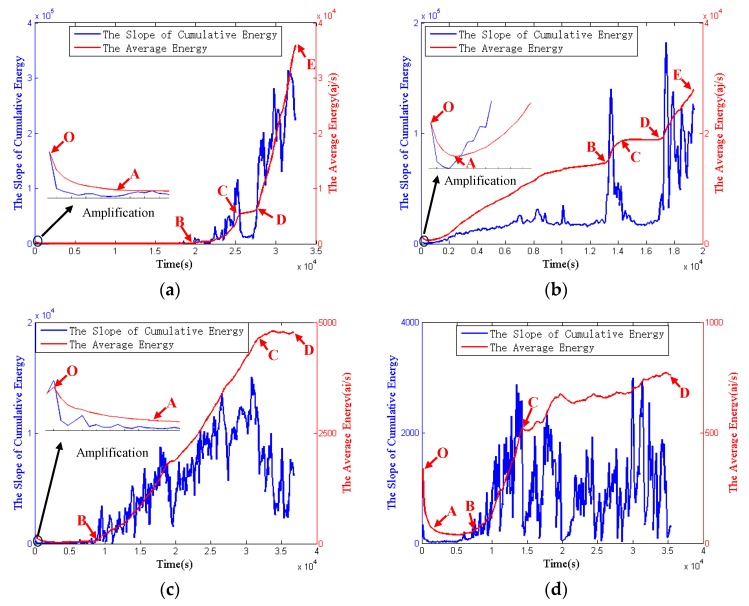
The cumulative energy and the slope of cumulative energy: (**a**) the first specimen; (**b**) the second specimen; (**c**) the third specimen; (**d**) the fourth specimen.

Figure 4 shows the average energy and the slope of cumulative energy, over time, of the four specimens. Comparing the tendencies of the average energy in four specimens, it is obvious that the first and the second specimens have similar tendency, and they both have one descent phase (the OA phase), two slower rise phase (the AB phase and the CD phase) and two faster rise phases (the BC phase and the DE phase). However, the third and the fourth specimens just have one descent phase (the OA phase), two slower rise phase (the AB phase and the CD phase), and one faster rise phase (the BC phase). The tendencies of the third specimen and the fourth specimen are similar with the OBD phases of the first and the second specimens. Furthermore, the descent phases in four specimens mean the energy and the growth rate of energy in the initial stage of the experiment is low.

**Figure 5:**
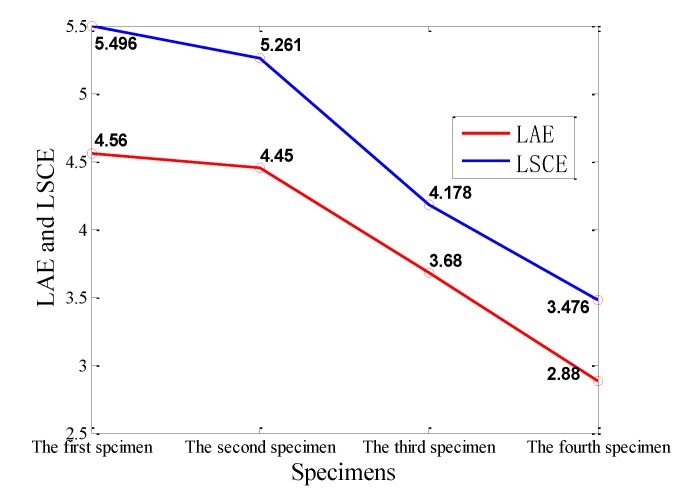
The results of the max LAE (logarithm of the average energy) and the max LSCE (logarithm of the slope of cumulative energy) in four specimens.

Figure 5 shows the max LAE and the max LSCE in four specimens. According to the value of the max LAE, the four specimens can be divided into three groups based on different values of the max LAE. The first group includes the first specimen and the second specimen, and their max LAE are similar (4.56 and 4.45, respectively). The second group includes the third specimens, whose max LAE is 3.68. The third group includes the fourth specimen, and its max LAE is 2.88, which is the minimum in four specimens. Meanwhile, similar to the max LAE, the results of four specimens can also be divided into three groups, according to different values of the max LSCE. The first group includes the first and the second specimens whose values are around 5, and the second group includes the third specimen whose value is 4.178. The third group includes the fourth specimen whose value is 3.476. It is obvious that the tendencies of the max LAE and the max LSCE are decreasing from the first specimen to the fourth specimen. This result may mean that the wear degree of four specimens gradually becomes severe from the fourth specimen to the first specimen, which will be discussed in Section 3.2 and Section 4. Furthermore, it can be summarized that when its max LAE is above 4, and its max LSCE is above 5, it belongs to first group; when its max LAE is between 3 and 4, and its max LSCE is between 4 and 5, it belongs to second group; when its max LAE is below 3 and its max LSCE is below 4, it belongs to third group.

### 3.2. Analyses Based on Micrographs and Surface Roughness

**Figure 6:**
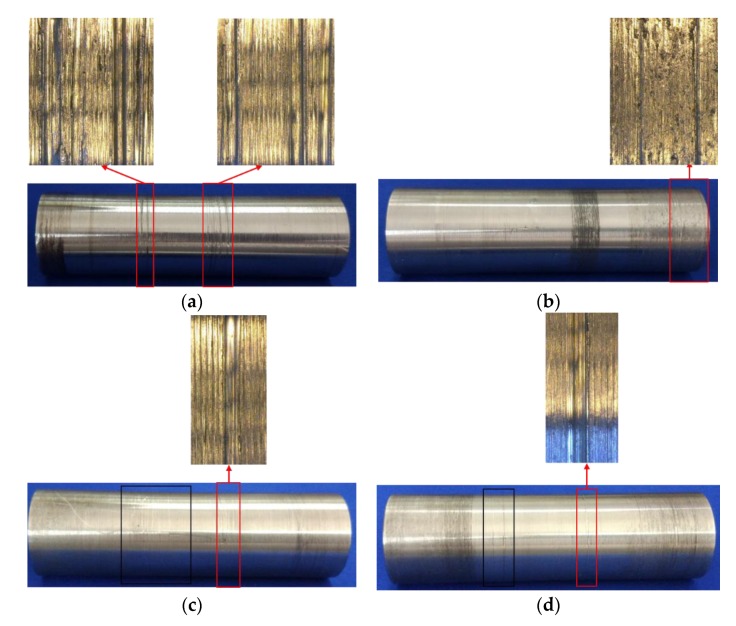
Worn micrographs of four specimens: (**a**) the first specimen; (**b**) the second specimen; (**c**) the third specimen; (**d**) the fourth specimen.

Figure 6 shows the micrographs of specimens after the tests. It is obvious that third and the fourth specimens both have one conspicuous groove and some shallow scratches, which means abrasive wear occurs on the surface [73], however, the number of shallow scratches of the third specimen is more than that in the fourth specimen (as shown in the black rectangle in Figure 6c,d). By contrast, the conspicuous grooves of the first and the second specimens are more than 1, and the first specimen has the most numbers of grooves in all four specimens. In addition, the trails of transfer particles adhering to the surface of the first and the second specimens can be seen, which means adhesive wear occurs on the surface [73]. According to Ref. [74], the adhesive wear is more severe than the abrasive wear, therefore, the wear degree of the first and the second specimens is more severe than the third and the fourth specimens.

Furthermore, the surface roughness in wear area of four pins (the areas are enclosed by red lines in Figure 6) are shown in Table 4, and it can be found that the maximum roughness and the average roughness of four specimens are larger than that of the control specimens, which means wear occurred in the surface of four specimens. Compared the roughness of four specimens, it is obvious that the maximum roughness and the average roughness gradually increases from the fourth specimen to the first specimen, which is in good agreement with the tendency of the LAE and the LSCE. Obviously, the wear degree of four specimens gradually becomes severe from the fourth specimen to the first specimen, and the wear degree of the first specimen is the most severe among the four specimens, which confirms the speculation in Section 3.1. Therefore, the values of the max LAE and the max LSCE can reflect the wear degree of pin connections, and different values of the max LAE and the max LSCE represent different wear degrees. Larger values of the max LAE and the max LSCE mean more severe wear.

### 3.3. Features of b-Value Method and Ib-Value Method

**Figure 7:**
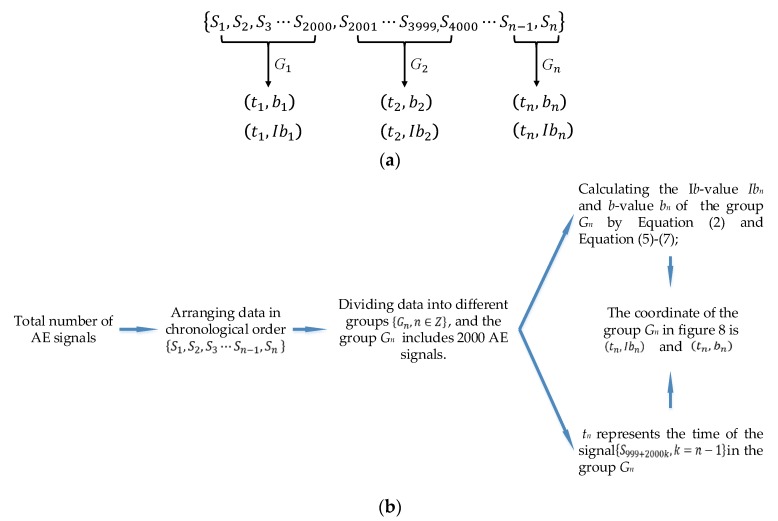
The flow chart of *b*-value method and *Ib*-value method: (**a**) the process of dividing signals into different groups; (**b**) the calculating process.

**Figure 8:**
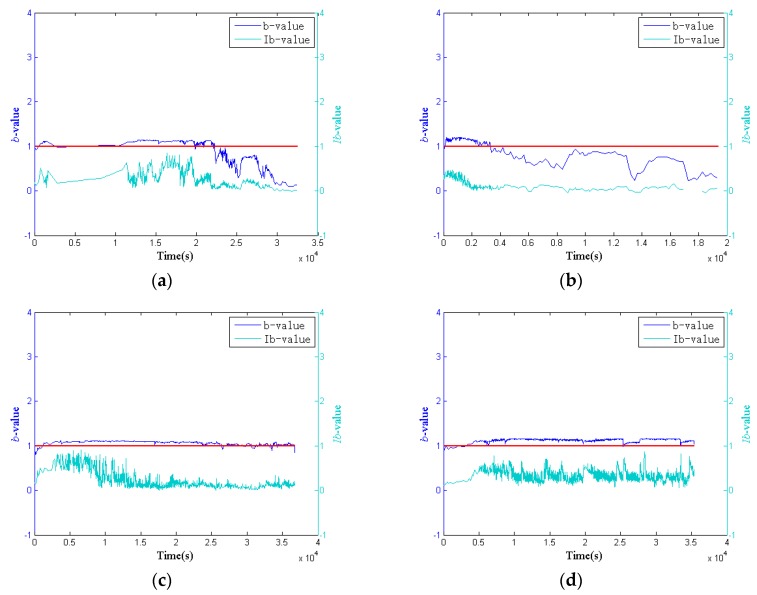
*b*-value and *Ib*-value of four specimens: (**a**) the first specimen; (**b**) the second specimen; (**c**) the third specimen; (**d**) the fourth specimen.

**Figure 9:**
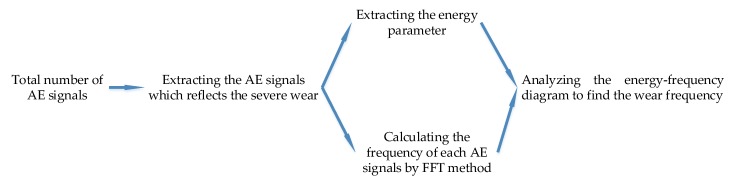
The flow chart of frequency-based analysis.

In this paper, the total number of AE signals in a chronological order $\left\{S_{1},\;S_{2},S_{3}\cdots S_{n-1},S_{n}\right\}$, and 2000 AE signals, were divided into groups which are defined as *G*_1_, *G*_2_, …, *G_n_* (as shown in Figure 7). The *b*-value *b_n_* and the *Ib*-value *Ib_n_* of the group, *G_n_*, can be calculated by using Equation (2) and Equations (5)–(7). In the group *G_n_*, the signal *S_n_* has its own occurring time *T_n_*, and this paper chose the occurring time $\left\{T_{999+2000k},k=n-1\right\}$ of $\left\{S_{999+2000k},k=n-1\right\}$ to represent the occurring time of the group *G_n_*. This occurring time $\left\{T_{999+2000k},k=n-1\right\}$ is defined as *t_n_*. Therefore, the coordinate of the group *G_n_* in Figure 8 is $\left(t_{n},b_{n}\right)$ and $\left(t_{n},Ib_{n}\right)$. The detailed process is illustrated in Figure 7 and Figure 9. The results have been plotted in Figure 8. The blue lines represent *b*-value curves, the green lines represent *Ib*-value curves, and the red lines represent the value of one. Comparing the *b*-value curves of four specimens, it can be found that the curves in the first and the second specimens fluctuate in the range of −1 to 3, and that in the third and the fourth specimens fluctuate around 1 and 2 (as illustrated in Table 5), respectively. By contrast, the *b*-value curve of the fourth specimen is not only smoother, but also higher than the red line. In addition, at the second half of *b*-value curves in the first and the second specimens, the *b*-value is much lower than the red line. The *b*-value curve of the third specimen is like a transitional curve: a small section is slightly lower than the red line at the beginning, however, it is higher than the red line in other sections. Meanwhile, the range of *Ib*-value curves of four specimens are 0 to 1, and the curves of four specimens are steadier than the *b*-value curves, which makes it be difficult to distinguish the wear degree of four specimens. Therefore, compared with *Ib*-value method, the *b*-value method is preferable to distinguish the wear degree of pin connections.

### 3.4. Frequency Spectrum of Wear

Results of the wear micrographs of four specimens reveal that severe wear occurred in the first and the second specimens, therefore, waveforms of the first and the second specimens can be used to analyze the frequency spectrum of severe wear. It can be found that when severe wear happens, the energy value of specimens is larger than 10,000 aJ. Therefore, this paper analyzes the frequency spectra of AE signals whose energy value is larger than 10,000 aJ in the first and the second specimens.

**Figure 10:**
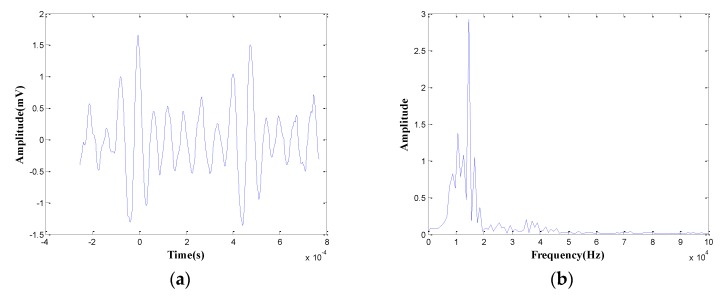
The waveform diagram and the spectrogram diagram of one hit: (**a**) the waveform diagram; (**b**) the spectrogram diagram.

**Figure 11:**
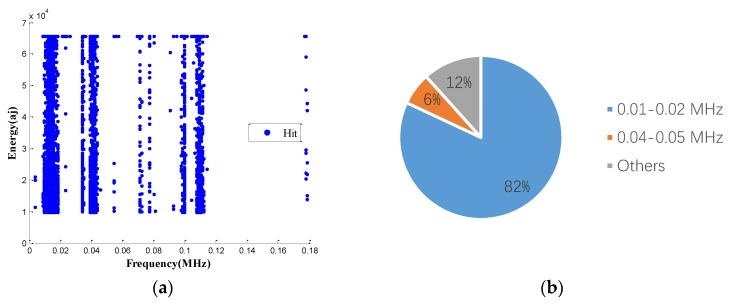
The frequency distribution of the first specimen: (**a**) frequency spectrum (energy > 10,000 aJ); (**b**) proportion of different frequency.

**Figure 12:**
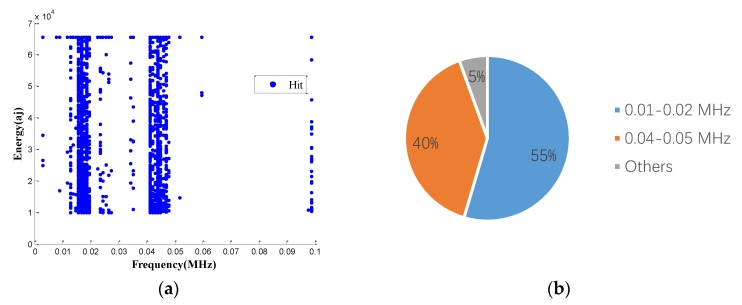
The frequency distribution of the second specimen: (**a**) frequency spectrum (energy > 10,000 aJ); (**b**) proportion of different frequency.

**Figure 13:**
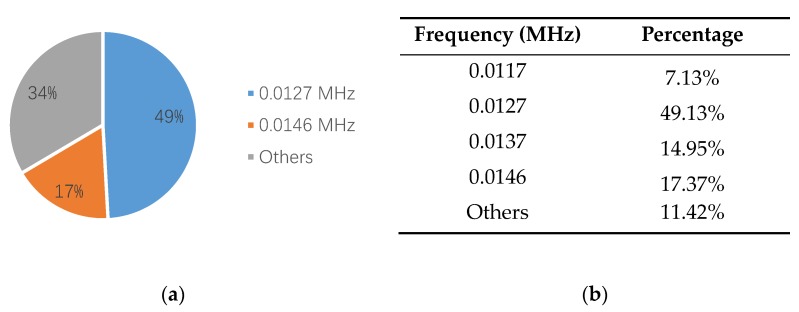
The frequency proportion between 0.01–0.02 MHz in the first specimen: (**a**) pie chart; (**b**) table.

**Figure 14:**
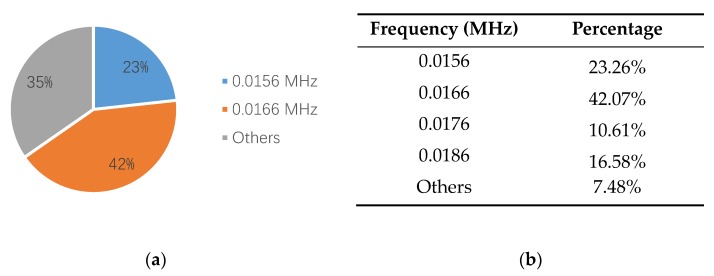
The frequency proportion between 0.01–0.02 MHz in the second specimen: (**a**) pie chart; (**b**) table.

Figure 10a is a waveform diagram of one hit, and the energy is the integral of the rectified voltage signal over the duration of the AE hit (or waveform). Using the time domain waveforms, the frequency information can be obtained by the FFT method. Figure 10b is the spectrogram of the hit, and the main frequency can be obtained from the figure. Comparing the energy and the main frequency of hits, the wear frequency can be found. The detailed analysis process is shown in Figure 10. The results are shown in Figure 11 and Figure 12. It is obvious that the wear frequency spectra in the low frequency range of the first and the second specimens mainly distribute in the range of 0.01 to 0.02 MHz. More detailed information of the wear frequency spectra is shown in Figure 13 and Figure 14, which clearly reveal that the main frequencies in the first and the second specimens are different. In the first specimen, the main frequency in the low frequency range is around 0.0127 MHz, however, this is around 0.0166 MHz in the second specimen. The results of wear photos show that the wear degree of the first specimen is more severe than that in the second specimen, therefore, the main wear frequency may decrease with the increase in wear degree in the low frequency range for the pin connections.

## 4. Discussion

**Figure 15:**
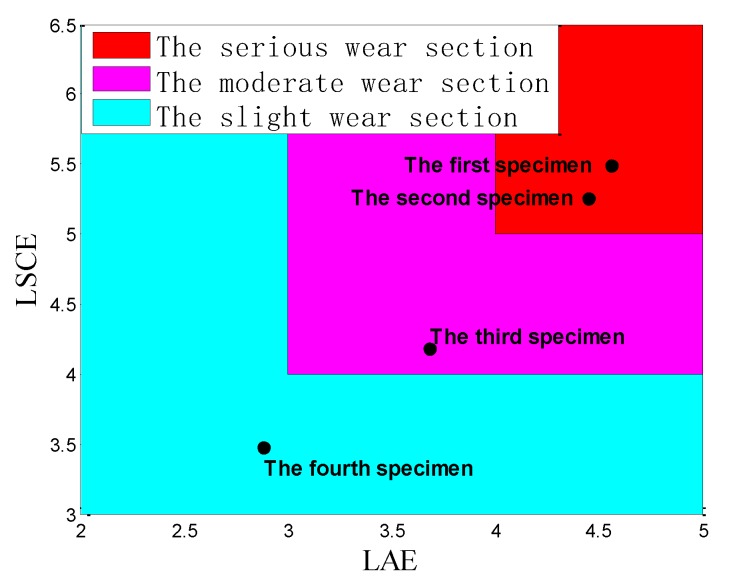
Quantification of wear degree in four specimens.

The severe wear occurring on the surface of pin connections may result in severe damage in structure, however, the wear process of pin connections is complicated, and can be influenced by various factors, such as loads, roughness of surface, lubrication condition, and others. This paper explores the feasibility of quantifying the wear degree of pin connections and setting a criterion to detect severe wear. The analysis of Section 3.1 and Section 3.2 have demonstrated that the wear degree can be reflected by the max LAE and the max LSCE. Referring to the results of four specimens that were divided into three groups based on different values of the max LAE and the max LSCE in the Section 3.1, it is obvious that three groups represent different wear degrees: the first group represents severe wear; the second group represents moderate wear; and the third group represents slight wear. Therefore, the wear degree of pin connections can be quantified by the values of max LAE and the max LSCE: when its max LAE is above 4 and its max LSCE is above 5, it is severe wear; when its max LAE is between 3 and 4 and its max LSCE is between 4 and 5, it is moderate wear; when its max LAE is below 3 and its max LSCE is below 4, it is slight wear, as shown in Figure 15.

**Figure 16:**
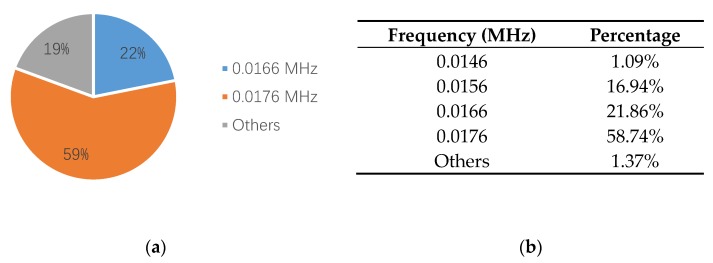
The frequency proportion between 0.01–0.02 MHz in the third specimen: (**a**) pie chart; (**b**) table.

**Figure 17:**
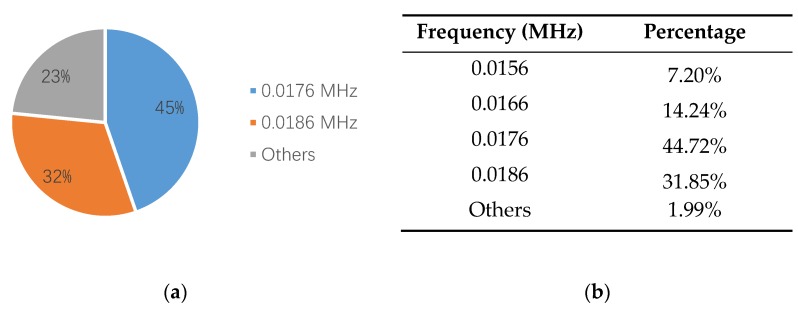
The frequency proportion between 0.01–0.02 MHz in the fourth specimen: (**a**) pie chart; (**b**) table.

Furthermore, to confirm the prior predictions in the end of the Section 3.4, the proportions of different frequencies in 0.01 to 0.02 MHz, in the third and the fourth specimens, are counted in Figure 16 and Figure 17. Since the number of AE signals—whose energy value is large—is small, to balance the energy value of AE signals and the number of AE signals, the energy values of AE signals, which were selected for spectrum analysis, are larger than 1600 aJ and 200 aJ, in the third and the fourth specimens, respectively. It can be found that the main frequency of the third and the fourth specimens are the same, which is 0.0176 MHz. The tendency of the main frequency between 0.01 to 0.02 MHz, from the first specimen to the fourth specimen, is rising, which is opposite of the tendency of the wear degree from the first specimen to the fourth specimen. This verifies the prior prediction: the main wear frequency decreases with the increase of the wear degree in the low frequency range for the pin connection studied in this paper.

On the other hand, literature studies [40,75,76] have proposed that the value “1” can be as the criterion to judge whether severe damage occurs in concrete beams. When the *b*-value is lower than 1, severe damage occurs in a structure. This paper applied this criterion to detect severe wear occurring on the surface of pin connections. The results show that severe wear occurred in the first and the second specimens according to this criterion, which is consistent with the results of the results of Section 3.2. Therefore, the value “1” can used be as the criterion to detect severe wear. The above analysis verifies that the value “1” can be used as a criterion to detect severe wear in pin connections.

## 5. Conclusions

The lack of a simple and reliable method in the detecting of the wear degree of pin connections motivated this research work. In this paper, using the max LAE and the max LSCE method, and the *Sb-*value method, to quantify the wear degree of pin connections based on AE signals, was proposed. An apparatus to conduct the accelerated pin connections’ wear test was designed and fabricated. The AE sensor was mounted on the test apparatus in a nondestructive way, and was capable of real-time monitoring. The experimental results show that the wear degree of pin connections can be divided into three wear degrees by different values of the max LAE and the max LSCE, as shown in Figure 15. The results of the *b*-value method and the *Ib*-value method indicate that the *b*-value method is preferable to distinguish the wear degree of pin connections, and the *b*-value method reveals that the value “1” can be applied as a criterion to classify the wear as severe for a pin connection. Furthermore, this research confirms that the wear frequency spectrum in the low frequency range mainly concentrates in the range of 0.01 to 0.02 MHz for the type of pin connections studied in this paper. Further analyses of this wear frequency spectrum found that the main wear frequency decreases with the increase of the wear degree in the low frequency range for the pin connection. In summary, the research demonstrates that the wear degree can be quantified by the max LAE and the max LSCE method, and the state of severe wear can be judged by the *b*-value method, all using AE signals. Further research will involve the use of the improved *b*-value relationship and the study of relationship between the roughness of wear surface and the AE signals. In future, we will also explore the modeling of AE signals generated from a pin connection during its wear process by using the fractal contact theory [77,78].

## Figures and Tables

**Table 1:** Material composition of specimens.

Elements	Proportion
C	≤0.07
Mn	≤2.00
P	≤0.035
S	≤0.030
Si	≤1.00
Cr	17.00~19.00
Ni	8.00~11.00

**Table 2:** Detailed parameters of VHX-600E digital microscope.

Parameters	Range
Magnification	20× to 200×
Observation range	19.05–1.14 mm
Repeat position precision	±0.5 µm

**Table 3:** Detailed parameters of PGI 840 roughmeter.

Parameters	Range
Measurement length	120 mm
Movement speed	0.1/0.25/0.5/1.0/10.0 mm/s
Measurement speed	0.1/0.25/0.5 mm/s
Sampling interval in horizontal	0.15 μm/0.1–15 mm 0.25 μm/15–30 mm 1 μm/30–200 mm
Accuracy of main spindle	±(0.02 μm+0.0003 μm)

**Table 4:** The surface roughness of four specimens.

Specimens	The Average Surface Roughness (μm)	The Maximum Surface Roughness (μm)
The control specimen	0.8473	1.1247
The first specimen	6.5552	8.4731
The second specimen	5.1232	6.9239
The third specimen	4.6801	5.6099
The fourth specimen	4.7704	5.2681

**Table 5:** The statistic of *b*-value distribution.

**Specimens**	**The Number of Data Whose *b*-Value Is Lower than 1**	**Total Number of Data**	**Proportion**
The first specimen	307	863	35.57%
The second specimen	415	415	26.98%
The third specimen	53	2176	2.44%
The fourth specimen	18	2943	0.61%
